# Supplementary material for: Patterns of Intron Gain and Loss in Fungi
Source: PLoS Biol. 2004 Nov 30;2(12):e422. doi: 10.1371/journal.pbio.0020422 (PMC532390; doi:10.1371/journal.pbio.0020422)
Supplement: Table S1 — Also available at http://genes.mit.edu/NielsenEtAl/. (4.3 MB ZIP). [file pbio.0020422.st001.zip › NielsenEtAl/html/842.html]

AN6933.1.NCU02906.1.MG08562.1.FG00805.1


```
 CLUSTAL W (1.82) Multiple Sequence Alignments - Introns Inserted


Sequence 1: NCU02906.1	611 aa
Sequence 2: MG08562.1	591 aa
Sequence 3: FG00805.1	662 aa
Sequence 4: AN6933.1	581 aa
Alignment Length: 672 aa
Number Identitical Residues: 269 aa
Alignment Score (without introns) 14199


MG08562.1 	-----------------------------------MP----------SKFAHLPLSTSGP
NCU02906.1	----------------------MSHSQNPQDEQAQKG----------SKFSHLPLSTSGP
FG00805.1 	MFKLTKVRSSRVYRFCSSPLLPMRSLNNNYATQAEKSQSTTTKNKSNSKYENLPLSTSGP
AN6933.1  	--------------------------------MS------------LARFKHLPRATQGP
          	                                 :             ::: :** :*.**

MG08562.1 	LDCALSGTAILNHPFFNKGSAFTAAERRDFGLLGLLPQNIQTLEQQANRAYAQYSSRPDD
NCU02906.1	LECALKGTVLLNHPYFNKGSAFTKEERRDFALHGLLPPQIQSLEQQVQRAYEQYCSQPND
FG00805.1 	INCALSGTSLLNTPYLNKGSAFPPDERRAFNLTGLLPQGVQTLEQQCKRAYEQYSSRPDD
AN6933.1  	IKCPYKGAALLGNANYNKGSAHSERERREFNLHGLLPPNIQTLDEQVERAYQQYKSRPDD
          	:.*. .*: :*. .  *****..  *** * * ****  :*:*::* :*** ** *:*:*

MG08562.1 	LAKNTFLTSLKEQNEVLFYR~-----------------LLHDNLPEMFGVVYTPTEGDAI
NCU02906.1	LAKNTFMTSMKEQNEVLFYR0-----------------LLHDHLDEMFSVVYTPTEGEAI
FG00805.1 	LAKNTFLTSLKDQNEVLYYK~ASYLPACHEGITDCQIQLIQTHLDEMMSIVYTPTEGDAI
AN6933.1  	LAKNTFMASMKSQNQVLYYR~-----------------LLQTHLKEMFSVIYTPTEADAI
          	******::*:*.**:**:*:                  *:: :* **:.::*****.:**

MG08562.1 	QNYSRLFRRPEGCFLNVNDV--DRVREDLQRWGRPEDVDYIVVSD~GE-~-EILGIGDQG
NCU02906.1	QNYSRLFRRPEGVFLNINDM--DSVKRDLAQWGKPEDIDYIVVTD1GE-~-EILGIGDQG
FG00805.1 	QNYSRLFRRPEGCYLNIQDP--DHVEHNLAQWGNPEDIDYIVVSD1GE-~-EILGIGDQG
AN6933.1  	QNYSRLFRKPEGCYLSIRDHGEKEIDECFANFSGGDDVDYIVVSD~GEQ0WQILGIGDQG
          	********:*** :*.:.* ... : . : .:.  :*:*****:* **.  :********

MG08562.1 	CGGILISIAKLVLTTLCAGLHPNRTLPVVLDCGTDNQGLLDDPLYLGLRERRVRGEKYDS
NCU02906.1	CGGILISIAKLVLMTICGGIHPNRVLPVVLDCGTNNEELLKDDLYLGLRQKRARGDKYDD
FG00805.1 	VGGILISVAKLVLTTLCAGVHPNRTLPVVLDCGTDNKELLEDELYLGLKEKRVRGEEYAK
AN6933.1  	VGAILISVAKLVITTACAGIHPSRQLPVVLDCGTNNEELLNDKLYLGLRQRRAQGEEYDK
          	 *.****:****: * *.*:**.* *********:*: **.* *****:::*.:*::* .

MG08562.1 	LVREFVAAARDLFPRAVIHF~--EDFGVTNA~RRILDTYRPRAPVFNDDVQGTGCVVLAA
NCU02906.1	FVETFIKAARELYPRAYIHF~--EDFGLDNA1RRLLERYRPQMPCFNDDVQGTGCVTLAA
FG00805.1 	FVDTFVQSARKLYPNAYIHF~--EDFGLGNA1RKILERYRPDFACFNDDVQGTGCVTLAA
AN6933.1  	FVDKFVRMARKRFPNAYIHL2CSEDFGLQNA~KRILDRYRSQLPCFNDDIQGTGCVTLAA
          	:*  *:  **. :*.* **: .:****: ** :::*: **.  . ****:******.***

MG08562.1 	IMAGMHVAGLKIQDLRMVVFGAGSAGTGIADMVRDAIAEESGCGKDCAAEQIW~LVDKPG
NCU02906.1	IMAGLHISKQKLSDVRMVVFGAGTAGVGIADHVRDAIAADADISPEEAGKQIW2LIDKPG
FG00805.1 	ILSGLHASDQKLKDLRMVVFGSGSAGLGIADQVRDAIATEGNMSHEDAANQIW2LIDKSG
AN6933.1  	LMAGLHVSNVKLKDVRVVCYGSGSAGTGIADQISDAIATEAGFSKSDALKQIW2CIDKQG
          	:::*:* :  *:.*:*:* :*:*:** **** : **** :.. . . * :***  :** *

MG08562.1 	LLT-TATQN-DTAQTPYAKKP--WGEGHGTDLLAVVDEVRPNVLIGTSTVPGAFTEEIVR
NCU02906.1	LLT-TKSESLSDAQKKYAKSHDDWAN-EKTDLLAVIKEVKPHILVGTSTKPKSFTEEVVR
FG00805.1 	LLT-SETEDVTESQKMYIKDVSEWKD-KDADLLSVVKEIRPNVLVGTSTKAGAFTEDVIR
AN6933.1  	LLLKSQGDALTASQKYFAKEDNEWPEGQDIDLYSVIKHVKPHVLIGTSTKPGSFTEETIR
          	** .:  :    :*. : *. ..* :..  ** :*:..::*::*:**** . :***: :*

MG08562.1 	AMAKHAPRPIIMPLSNPTRLHEAVPADLMAWTDGKALVATGSPFAPVKLDGGR-----EV
NCU02906.1	EMAKHVERPIILPLSNPTKLHEAVPEDLLNWTDGKALVATGSPFDPVKGPWGKDGKEIEI
FG00805.1 	AMTEHHERPIVLPLSNPTRLHEAKPEDILNWTDGKALVATGSPFPAVKGPWGKNGEEITI
AN6933.1  	EMAKHVDRPIIFPLSNPTRLHEAQPQDITKWTDGKALIATGSPFPPVEYNGTKT----EI
          	 *::*  ***::******:**** * *:  *******:****** .*:    :      :

MG08562.1 	E~IAECNNSVVFPGIALGSVLSRARLVTDSMLVGAVKAVAAMSPALKDPAGSLLPGVDEV
NCU02906.1	D~VAECNNSVVFPGIGLGSVLSRANRVTDKMLMAASKGVAEMGPALRDDTAPLLPGVDVV
FG00805.1 	N~VGECNNSVVFPGIGLGCILSRAKHLTDRMLVAAVEGVSSLSPVLKDSTAPLLPDVGSV
AN6933.1  	V1SAECNNSTAFPGIGLGAVLSRASRLSEKMVVAASKALAAKAPALEDPNKPLLPDVENV
          	   .*****..****.**.:****  ::: *::.* :.::  .*.*.*   .***.*  *

MG08562.1 	RNVSVAVASGVIRAAVQEGVATEEGIPRGKDDGDLEEWVREQMWNPVYRELRKVGGGDAT
NCU02906.1	RDVSVRVARRVILAAMEEGVVEEKDVPTNEED--LDEWIREQMWDPVYRPLKYVEGNKAS
FG00805.1 	RDVSVRIARNVIQAAVKDGEATQEGIPEDEED--LEEWIKEQMWDPEYRPLKQVDLESAT
AN6933.1  	RELSLNVAKAVIQTAVKEGLAQEEGIPEDEKD--LEDWIRAQMWEATYRDLEKAD-----
          	*::*: :*  ** :*:::* . ::.:* .:.*  *::*:: ***:. ** *. .      

MG08562.1 	REAKGELKQAGTLEG----
NCU02906.1	RGAKGDLRVLGNPNDVE--
FG00805.1 	REARGELKKAGTVHRTGHT
AN6933.1  	-------------------
          	
```
